# Behavioral Activation–Based Digital Smoking Cessation Intervention for Individuals With Depressive Symptoms: Randomized Clinical Trial

**DOI:** 10.2196/49809

**Published:** 2023-11-01

**Authors:** Jennifer Dahne, Amy E Wahlquist, Jacob Kustanowitz, Noelle Natale, Margaret Fahey, Evan M Graboyes, Vanessa A Diaz, Matthew J Carpenter

**Affiliations:** 1 Department of Psychiatry and Behavioral Sciences Medical University of South Carolina Charleston, SC United States; 2 Hollings Cancer Center Medical University of South Carolina Charleston, SC United States; 3 Center for Rural Health Research East Tennessee State University Johnson City, TN United States; 4 MountainPass Technology Chevy Chase, MD United States; 5 Department of Otolaryngology-Head and Neck Surgery Medical University of South Carolina Charleston, SC United States; 6 Department of Family Medicine Medical University of South Carolina Charleston, SC United States

**Keywords:** smoking cessation, depression, digital health, decentralized trial, mental health, depressive, RCT, randomized, controlled trial, smoking, smoke, smoker, quit, quitting, cessation, digital health, eHealth, e-health, NRT, nicotine, mobile health, mHealth, app, apps, application, applications

## Abstract

**Background:**

Depression is common among adults who smoke cigarettes. Existing depression-specific cessation interventions have limited reach and are unlikely to improve smoking prevalence rates among this large subgroup of smokers.

**Objective:**

This study aimed to determine whether a mobile app–based intervention tailored for depression paired with a mailed sample of nicotine replacement therapy (NRT) is efficacious for treating depression and promoting smoking cessation.

**Methods:**

A 2-arm nationwide remote randomized clinical trial was conducted in the United States. Adults (N=150) with elevated depressive symptoms (Patient Health Questionnaire-8≥10) who smoked were enrolled. The mobile app (“Goal2Quit”) provided behavioral strategies for treating depression and quitting smoking based on Behavioral Activation Treatment for Depression. Goal2Quit participants also received a 2-week sample of combination NRT. Treatment as usual participants received a self-help booklet for quitting smoking that was not tailored for depression. Primary end points included Goal2Quit usability, change in depression (Beck Depression Inventory-II) across 12 weeks, and smoking cessation including reduction in cigarettes per day, incidence of 24-hour quit attempts, floating abstinence, and 7-day point prevalence abstinence (PPA).

**Results:**

In total, 150 participants were enrolled between June 25, 2020, and February 23, 2022, of which 80 were female (53.3%) and the mean age was 38.4 (SD 10.3) years. At baseline, participants on average reported moderate depressive symptoms and smoked a mean of 14.7 (SD 7.5) cigarettes per day. Goal2Quit usability was strong with a mean usability rating on the System Usability Scale of 78.5 (SD 16.9), with 70% scoring above the ≥68 cutoff for above-average usability. Retention data for app use were generally strong immediately following trial enrollment and declined in subsequent weeks. Those who received Goal2Quit and the NRT sample reported lower mean depressive symptoms over the trial duration as compared to treatment as usual (difference of mean 3.72, SE 1.37 points less; *P*=.01). Across time points, all cessation outcomes favored Goal2Quit. Regarding abstinence, Goal2Quit participants reported significantly higher rates of 7-day PPA at weeks 4 (11% vs 0%; *P*=.02), 8 (7-day PPA: 12% vs 0%; *P*=.02), and 12 (16% vs 2%; *P*=.02).

**Conclusions:**

A mobile app intervention tailored for depression paired with a sample of NRT was effective for depression treatment and smoking cessation. Findings support the utility of this intervention approach for addressing the currently unmet public health treatment need for tailored, scalable depression-specific cessation treatments.

**Trial Registration:**

ClinicalTrials.gov NCT03837379; https://clinicaltrials.gov/ct2/show/NCT03837379

## Introduction

Despite significant declines over the last 50+ years in cigarette smoking prevalence among the general population, smoking rates remain astonishingly high among certain vulnerable subgroups. One such subgroup is the 48 million US adults with depressive symptomatology [1]. Smoking prevalence estimates among those with depressive symptoms range between 20% and 50% [2-6], and these symptoms are a robust risk factor for the etiology of tobacco use across the lifespan [7-9]. Despite similar levels of motivation to quit [10,11] and incidence of quit attempts [12] as their nondepressed counterparts, depressed smokers are 30%-50% less likely to be abstinent one-month post quit [12], regardless of depression severity [13].

Tailored cessation treatments that incorporate depression-specific psychosocial treatment components increase long-term quit rates by up to 50% among those with current depressive symptoms [14]. However, these treatments are predominantly delivered via individual or group psychotherapy and thus have limited reach [15-18]. Digital health technologies offer an ideal strategy to further disseminate evidence-based depression-specific cessation treatment [19]. Smartphones and mobile apps are ubiquitous, and recent estimates suggest that 85% of US adults own a smartphone [20]. While a number of studies have developed and evaluated digital health smoking cessation treatments [21-24], few have been specifically tailored to the unique needs of those with depressive symptoms.

An ideal depression-specific psychosocial treatment to adapt for digital delivery must be both evidence-based and simple so that the treatment can be easily understood via a self-guided digital platform. Behavioral activation (BA) is an evidence-based depression intervention that focuses on regular self-monitoring to (1) examine already occurring activities and (2) facilitate the incorporation of new activities consistent with individualized values or goals across life areas [25]. BA specifically targets the anhedonic component of depression, identified by the transdiagnostic emotional vulnerability framework as central to the comorbidity of depression and smoking [26]. BA has an extensive literature base for the treatment of depression [25,27-35] as well as demonstrated promise for smoking cessation [36,37]. However, to our knowledge, no randomized clinical trials have evaluated BA-based digital intervention approaches for smoking cessation among individuals who smoke cigarettes and have depressive symptoms.

Importantly, because medication management is a critical component of any tobacco cessation intervention [38], it would be unwise to provide a digital cessation intervention without also providing pharmacotherapy. Yet, providing a full course of medication runs counter to the scalable, hands-off advantages of digital interventions. Thus, an appropriate medication delivery strategy to pair with a scalable digital intervention must foster the translational potential of the intervention and connect patients to existing outlets for accessing cessation medications.

To address the lack of effective, scalable smoking cessation approaches for individuals with depressive symptoms, our team developed “Goal2Quit,” a BA-based self-directed mobile app intervention. Goal2Quit was paired with a short duration of mailed nicotine replacement therapy (NRT) with instructions provided in Goal2Quit that users should contact their primary care provider, their state quitline, or visit a local pharmacy to obtain additional NRT or other cessation medications. This nationwide decentralized randomized clinical trial aimed to examine the usability and uptake of Goal2Quit among adult smokers with depressive symptoms as well as its efficacy for improving depression and promoting smoking cessation.

## Methods

### Ethical Considerations

The study and protocol were approved by the Medical University of South Carolina institutional review board (Pro00074015). Participants provided electronic written informed consent before enrollment. Trial information is presented according to the CONSORT (Consolidated Standards of Reporting Trials) and the trial was preregistered on ClinicalTrials.gov (NCT03837379).

### Participants

Study participants were recruited nationwide via web-based advertising (2020-2022). Consistent with guidelines for preventing participant deception in web-based nicotine and tobacco research studies [39], study advertisements intentionally avoided advertising eligibility criteria to prevent respondents from being able to answer screening questions in a way that would make them appear eligible when not truly eligible. Study advertisements included a link to a brief REDCap (Research Electronic Data Capture) [40] eligibility screening. Eligible participants were English-speaking adults who currently smoked (self-report of ≥5 cigarettes per day [CPD] for at least 25 out of the last 30 days for at least the last 6 months), had elevated depressive symptoms (Patient Health Questionnaire-8≥10 [41]), and had been seen by a primary care provider in the last year. Participants were excluded if they had contraindications for NRT, had severe visual impairment that would limit the ability to use an app, or reported suicidality at baseline.

### Procedures

All study procedures were delivered fully remotely, consistent with a decentralized clinical trial design [42]. Following informed consent, enrolled participants completed baseline assessments via REDCap and then were randomized 2:1 to receive either Goal2Quit or treatment as usual (TAU). Those randomized to Goal2Quit received help downloading the app, were provided a brief scripted overview on its use, and were instructed to use the app regularly throughout the course of the study. Following the baseline visit, participants were emailed or SMS text messaged a link to complete follow-up assessments in REDCap weekly for 8 weeks with an additional final follow-up at 12 weeks. Participants were compensated up to US $140 via electronic gift codes for completion of all assessments. Participants randomized to Goal2Quit were not compensated for treatment engagement.

To minimize fraud [39], at screening, we used identifying information to detect duplicate screening entries and disqualified all duplicates from enrollment. Following enrollment, individual participant records were flagged for potential fraud if any of the following were met: (1) completion of any study follow-up assessment in <5 minutes, (2) inaccurate answers to validity check questions embedded throughout assessments, or (3) straight-lined responses (answering with the same response to all items) on any survey. If a participant was flagged for potential fraud at 3 study time points, they were withdrawn, not invited to complete additional assessments, and not included in the analytic sample.

### Interventions

#### Goal2Quit

**Figure 1:**
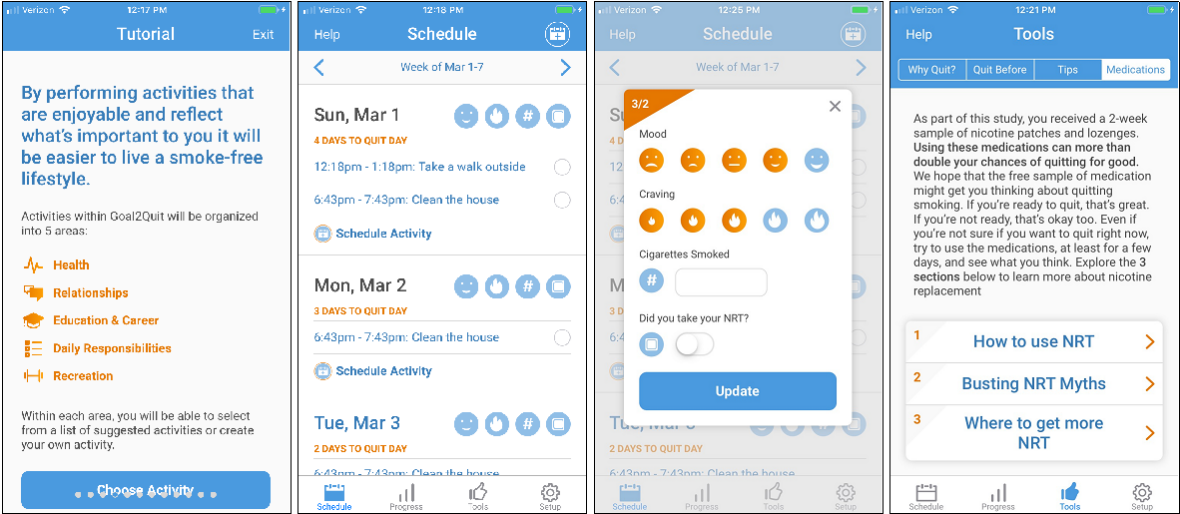
Goal2Quit. NRT: nicotine replacement therapy.

Goal2Quit treatment content is consistent with the original BA for smoking cessation group therapy manual [43], including both depression-specific and standard smoking cessation treatment components (Figure 1). Goal2Quit content is primarily interactive, with some text-based communication for providing psychoeducation, as well as push notifications to provide reminders of scheduled activities. When users first download Goal2Quit, they complete a psychoeducational tutorial illustrating the connection between negative mood and cigarette smoking, which highlights that Goal2Quit will help to improve mood in the service of promoting smoking cessation. Subsequently, users practice generating and scheduling activities across various life areas and set a quit date. Users then identify their most common triggers to smoke, select coping strategies for trigger management, and receive information about NRT provided as a part of study participation. After completion of the tutorial, Goal2Quit encourages users to regularly create, schedule, and complete new activities to improve mood. Standard smoking cessation treatment components including self-monitoring and relapse prevention strategies are embedded throughout the app.

Goal2Quit was paired with a mailed 2-week sample of NRT, inclusive of 14 mg nicotine patches and 4 mg nicotine lozenges. As nicotine lozenges were provided in addition to nicotine patches, we opted for uniform NRT patch dosing.

#### TAU

We selected TAU as an appropriate control since the central research question herein is whether Goal2Quit improves depression and smoking cessation outcomes relative to existing treatment services. Research staff provided participants with a digital copy of the National Cancer Institute’s “Clearing the Air: Quit Smoking Today” booklet [44], which provides evidence-based strategies for quitting smoking. “Clearing the Air” does not provide depression-specific treatment content, and TAU participants were not provided with NRT by the study.

### Assessments

#### Overview

All participants at baseline completed a general assessment of demographics as well as assessments of confidence in quitting smoking (0-10 Likert scale) [45] and motivation to quit smoking (0-10 Likert scale) [45]. Primary outcomes for this trial included measures related to (1) Goal2Quit usability and uptake, (2) depression, and (3) smoking cessation.

#### Goal2Quit Usability and Uptake

Assessment of Goal2Quit usability and uptake included self-reported app usability and passively collected app analytics. At week 4, participants randomized to the Goal2Quit condition completed the System Usability Scale [46], a 10-item assessment of a user’s experience interacting with a product or system. A score of 68 or higher is interpreted as above-average usability. Passive use data, captured across the 12-week trial period, included (1) total number of app sessions, (2) average time per session, (3) total time using the app, (4) total scheduled activities, and (5) total completed activities. Analytics data were used to determine app retention, defined as use within each week following download.

#### Depression

Participants self-reported depressive symptoms via the Patient Health Questionnaire-8 [41] at screening. Across the study follow-up period (including baseline), depressive symptoms were assessed via the Beck Depression Inventory-II (BDI-II) [47]. Scores on the BDI-II categorize depression severity as follows: 0-13=minimal depression, 14-19=mild depression, 20-28=moderate depression, and 29-63=severe depression. Worsening depression, defined as an increase of 10 or more points on the BDI-II from the baseline assessment or evidence of suicidality at any time point (response of “I would like to kill myself” or “I would kill myself if I had the chance” on the suicidal thoughts or wishes item of the BDI-II) were monitored throughout the course of the study for participants in both groups. In the event of worsening depression, participants were allowed to continue in the study but were provided with mental health resources (eg, therapy clinics) local to their area and encouraged to seek additional treatment.

#### Smoking Cessation

At each follow-up, participants self-reported (1) the number of cigarettes smoked per day over the last 7 days (also captured at baseline) and (2) the incidence of quit attempts lasting at least 24 hours since the prior assessment. Data regarding quit attempts were collapsed across time points to indicate any 24-hour quit attempt during the first 4, first 8, and all 12 weeks of follow-up. Data regarding CPD during the last week were coded as to whether the participant had reduced their mean CPD by at least 50% between baseline and week 4, week 8, and week 12. Those who reported any 7-day period of complete abstinence between baseline and week 12 were coded as having 7-day floating abstinence. At weeks 4, 8, and 12, those who reported smoking zero cigarettes over the last 7 days (prior to assessment) were coded as having 7-day point prevalence abstinence (PPA) for that time point. Cessation outcomes were based on self-report and were not biochemically verified.

### Data Analytic Plan

All individuals randomized to Goal2Quit or TAU and not removed for fraud were included in analyses. Descriptive statistics for baseline variables, usability metrics, and app uptake were calculated for the overall sample and per group, as appropriate. Between-group changes in depressive symptoms and CPD were compared via general linear models to account for repeated measures across time within an individual as well as the main effects of the baseline level of the outcome, group, time, an interaction between group and time, and baseline covariates determined a priori (sex, CPD, and depression). For nonsignificant interaction terms, the interaction term was removed from the model. For all modeling and analyses, an α=.05 level of significance was applied. Other smoking cessation outcomes (quit attempts and abstinence) were evaluated using logistic regression models to determine if there were group differences after adjustment for sex, baseline CPD, and baseline depression at the time point of interest. Logistic regression models were also used to evaluate any effect of app use on cessation outcomes within the Goal2Quit group. All analyses using binary outcomes were analyzed via an intent-to-treat approach, such that individuals with missing data were included in the analysis and treated as not having achieved the cessation outcome of interest. For analyses of continuous outcomes such as CPD and BDI, missing data were left as missing for that visit, with models including all visits completed by an individual.

## Results

### Participant Characteristics

**Figure 2:**
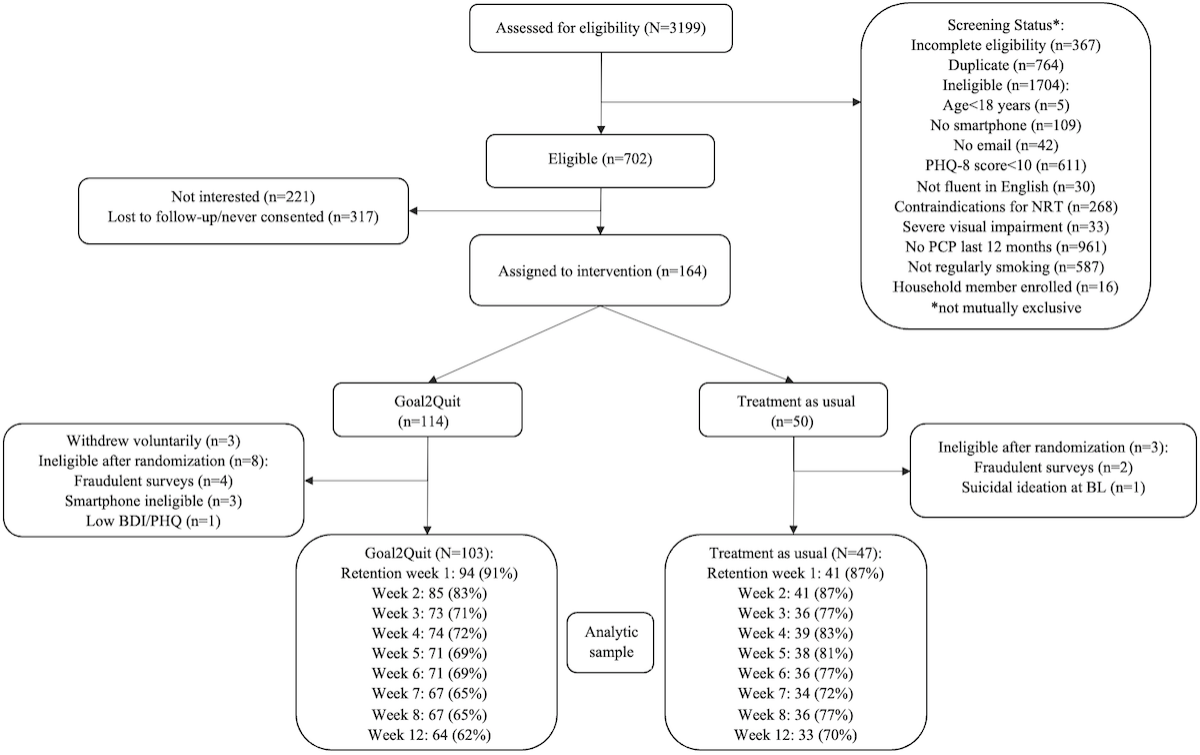
CONSORT diagram. BDI: Beck Depression Inventory; BL: baseline; NRT: nicotine replacement therapy; PCP: primary care provider; PHQ-8: Patient Health Questionnaire-8.

See Figure 2 for information on study screening rates, enrollment, allocation, and attrition, and Table 1 for demographics. There were no significant differences in study retention as a function of treatment group (Figure 2).

**Table 1:** Participant demographics.

Demographics		Full sample (N=150)	Treatment as usual (N=47)	Goal2Quit (N=103)
Age in years, mean (SD)		38.4 (10.3)	41.2 (11.2)	37.1 (9.7)
Sex (female), n (%)		80 (53)	27 (57)	53 (51)
**Race, n (%)**				
	Black	33 (22)	12 (26)	21 (20)
	White	100 (67)	26 (55)	74 (72)
	Other	17 (11)	9 (19)	8 (8)
Ethnicity (Hispanic or Latinx), n (%)		13 (9)	4 (9)	9 (9)
**Census region (based on state), n (%)**				
	Northeast	15 (10)	3 (6)	12 (12)
	Midwest	22 (21)	10 (21)	22 (21)
	South	94 (63)	31 (66)	63 (61)
	West	9 (6)	3 (6)	6 (6)
**Education, n (%)**				
	<High school diploma	54 (36)	19 (40)	35 (34)
	>High school diploma	96 (64)	28 (60)	68 (66)
**Annual household income in US** $**, n (%)**				
	<50,000	128 (85)	41 (87)	87 (84)
	≥50,000	22 (15)	6 (13)	16 (16)
**Type of smartphone, n (%)**				
	iPhone	49 (33)	13 (28)	36 (35)
	Android	101 (67)	34 (72)	67 (65)
Currently seeing a psychiatrist, therapist, or counselor (yes), n (%)		42 (28)	8 (17)	34 (33)
Currently participating in individual or group therapy (yes), n (%)		35 (23)	8 (17)	27 (26)
Ever prescribed medication for mental health (yes), n (%)		93 (62)	28 (60)	65 (63)
Current use of medication for mental health (yes), n (%)		43 (29)	13 (28)	30 (29)
Beck Depression Inventory-II baseline, mean (SD)		24.2 (10.2)	23.5 (10.6)	24.5 (10.0)
Baseline cigarettes per day, mean (SD)		14.7 (7.5)	14.4 (7.8)	14.8 (7.4)
Motivation to quit in the next month, mean (SD)		7.8 (5.3)	8.3 (8.7)	7.4 (2.5)
Confidence in quitting in the next month, mean (SD)		5.4 (2.6)	5.1 (2.7)	5.5 (2.6)

### Goal2Quit Usability and Uptake

The mean Goal2Quit usability on the System Usability Scale at week 4 was 78.5 (SD 16.9), with 70% scoring above the ≥68 cutoff for above-average usability [46]. There was considerable variability between participants regarding app engagement (Table S1 in Multimedia Appendix 1). Retention data for app use were generally strong immediately following trial enrollment and declined in subsequent weeks (Table S2 in Multimedia Appendix 2). NRT use was high with 76% (n=78) of Goal2Quit participants reporting use during the 12-week study period compared to only 17% (n=8) of the TAU group (*P*<.001).

### Depression

**Figure 3:**
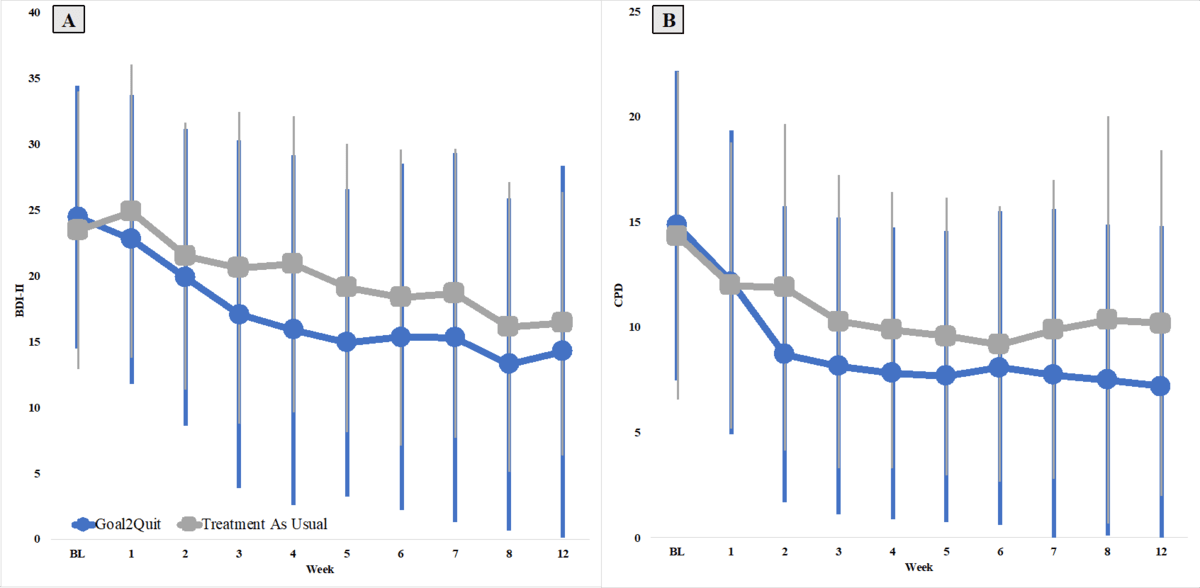
Change in (A) depressive symptoms and (B) CPD over time by treatment group. Raw means are depicted with corresponding SD. BDI-II: Beck Depression Inventory-II; BL: baseline; CPD: cigarettes per day.

There was no evidence that the association between group and change in depressive symptoms varied over time (*P*=.44). Both main effects of time and intervention were significantly related to change in depressive symptoms. On average, after adjusting for covariates and baseline BDI score, both groups showed a decrease in depressive symptoms over time (difference of mean 8.57, SE 0.86 points from week 1 to week 12; *P*<.001), but the Goal2Quit group reported lower average depressive symptoms over the course of the trial as compared to the TAU group (difference of mean 3.72, SE 1.37 points less for Goal2Quit; *P*=.01; Figure 3A).

Regarding worsening depression over time, 37 participants reported worsening mood, defined as an increase of 10 or more points on the BDI-II since baseline, at some point during the study duration (23 Goal2Quit, 14 TAU) and 11 participants endorsed suicidality (4 Goal2Quit, 7 TAU).

### Smoking Cessation

**Figure 4:**
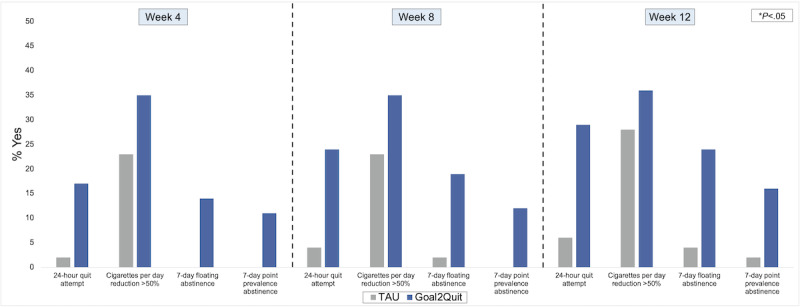
Smoking cessation outcomes as a function of treatment group. TAU: treatment as usual.

Across time points, all cessation outcomes favored Goal2Quit. Goal2Quit participants were more likely to have made a 24-hour quit attempt during the first 4 (17% vs 2%; *P*=.01), first 8 (24% vs 4%; *P*=.003), and across all 12 (29% vs 6%; *P*=.002) weeks of follow-up. Moreover, both floating and 7-day PPA were significantly higher for Goal2Quit participants relative to TAU at week 4 (Floating: 14% vs 0%; *P*=.01 and 7-day PPA: 11% vs 0%; *P*=.02), week 8 (Floating: 19% vs 2%; *P*=.01 and 7-day PPA: 12% vs 0%; *P*=.02), and week 12 (Floating: 24% vs 4%; *P*=.003 and 7-day PPA: 16% vs 2%; *P*=.02; Figure 4).

There was no evidence that the association between group and change in CPD varied over time (*P*=.08). Both time and intervention were significantly related to change in CPD. Overall, both groups decreased their CPD over the course of the study (difference of mean 4.71, SE 0.42 CPD less by week 12; *P*<.001), but the Goal2Quit group reported smoking less CPD than the TAU group (difference of mean 1.97, SE 0.93 CPD less; *P*=.03; Figure 3B).

Within the Goal2Quit group, total time spent using the app was significantly related to abstinence at week 12, with the odds of reporting 7-day PPA increasing 7% for every 5-minute increase in app use (odds ratio [OR] 1.07, 95% CI 1.01-1.13; *P*=.02).

## Discussion

These results preliminarily indicate that Goal2Quit paired with a mailed sample of NRT (1) is usable by adults with depressive symptoms who smoke cigarettes, (2) is efficacious for depression treatment, and (3) promotes smoking cessation. Regarding usability and treatment engagement, outcomes can be compared to other available digital interventions. A recent, also fully decentralized, trial evaluating 2 app-based smoking cessation interventions found that 42.9% of users continued to use their intervention beyond 1 week of download and only 14.6% beyond 4 weeks [48]. Comparatively, engagement with Goal2Quit was higher, with 68% of participants continuing to use the app beyond 1 week and 32% beyond 4 weeks. Other recent trials of digital health interventions for smoking cessation that include in-person trial components (rather than fully decentralized designs) have seen higher intervention engagement [49,50]. Thus, providing in-person support may help to facilitate higher levels of engagement with digital health interventions. Importantly, when evaluating engagement with digital interventions, indicators of optimal engagement may not mirror what would be used in traditional in-person interventions. Indeed, an advantage of digital interventions is that users can access treatment content in an on-demand fashion and thus may engage with the intervention for variable durations [51]. Users who engage with digital interventions for shorter periods of time may do so either because the intervention is not appealing to them or because they have benefited sufficiently, have incorporated the skills taught by the intervention into their lives, and thus no longer need to regularly engage with the digital intervention. In this trial, app engagement was highest early in the trial, which corresponded with the greatest decreases in symptoms of depression and CPD. These between-group differences then were sustained throughout the trial duration, consistent with the latter hypothesis, though additional research is needed to specifically evaluate these associations.

Regarding depression outcomes, Goal2Quit demonstrated efficacy for depression treatment. As compared to TAU, those who received Goal2Quit reported lower depression symptom severity over the course of the trial, with the most pronounced improvements occurring during the first 3 weeks after receiving the intervention, consistent with a prior in-person trial of BA for smoking cessation [36]. Although both groups experienced improvements in depression over time, those who received Goal2Quit reported consistently lower symptoms of depression across follow-up. Thus, Goal2Quit paired with an NRT sample may help to facilitate greater reductions in depression as compared to standard self-help materials for quitting smoking.

Regarding smoking cessation, on all indicators, Goal2Quit outperformed TAU. Those who received Goal2Quit were more likely to attempt to quit and achieve abstinence. Similar to depression outcomes, the greatest decreases in CPD occurred early in the trial, between baseline and week 2. While these decreases also mirror patterns of early app engagement, these changes in smoking behavior are also likely related to the duration of NRT (2 weeks) provided in combination with Goal2Quit. Cessation outcomes can be compared to those of standard, in-person BA-based group therapy for smoking cessation among individuals with depressive symptoms as well as other app-based interventions for broader groups of individuals who smoke cigarettes. Participants in a prior trial who received in-person BA in addition to 8 weeks of NRT patches had biochemically verified cessation rates of 28.6%, 17.1%, 11.4%, and 14.3% at 1, 4, 16, and 26 weeks post the quit date, respectively [36]. While cessation rates herein were lower, the expanded reach of a digital BA intervention must be balanced against improved efficacy for the more cumbersome in-person intervention. In-person, weekly therapy may confer better clinical outcomes, but for those who cannot access that modality, a self-directed, hands-off intervention like Goal2Quit could have an impactful effect on helping adults with depressive symptoms quit smoking while also improving depressive symptoms. As compared to other app-based approaches (not tailored for depression), self-reported cessation at 3 months herein was similar to prior trials (eg, 16% self-report 7-day PPA at 3 months for NCI’s QuitGuide app and 27.3% for the iCanQuit app [52]).

Given that the purported mechanism of BA is the completion of goal-driven activities, intervention refinements that specifically promote activity completion may help to bolster therapeutic effects in the future. On average, participants scheduled 72.8 activities across the 12-week study duration. This rate of activity scheduling is consistent with the original BA for smoking cessation treatment manual [43], which introduces activity scheduling during session 3, instructing patients to schedule 1 to 3 new activities, and then adds 1 additional new activity per week for the remaining 5 therapy sessions. Though rates of activity scheduling were comparable to what is typical during traditional forms of BA for smoking cessation therapy, rates of activity completion (14 on average per participant, ie, 19% of those scheduled) were low. It is unclear whether these activities were truly not completed or whether they were completed but were not marked as such in the app. Additional refinements to either promote completion of activities or to facilitate recording activities as completed when done may help to facilitate treatment response.

The results of this study should be interpreted with limitations in mind. We were unable to disentangle the effects of the Goal2Quit app versus the NRT sample since the intervention group received both. However, this was an a priori decision as digital cessation interventions must be paired with pharmacotherapy to address both behavioral and physical symptoms of nicotine dependence. Additionally, all cessation outcomes were based on self-report. In our team’s prior remote trials, biochemically verified abstinence rates have been somewhat lower than self-reported abstinence [53]. Finally, study generalizability may be limited only to those fluent in English who own a smartphone, and the durability of intervention effects beyond 12 weeks is unclear.

There are a number of additional important future directions stemming from this work. While digital interventions may increase treatment accessibility, sustainability must also be considered. Next steps should focus on the evaluation of effectiveness, implementation, and eventually embedding the intervention in clinical practice or other settings that can benefit [54]. Moreover, recognizing that engagement with Goal2Quit was variable and not all participants experienced clinical benefits, it will be important to develop methods to identify those who are most likely to respond to the intervention so that treatment can be tailored to the unique needs of each individual [55].

In conclusion, Goal2Quit paired with a brief, mailed sample of NRT was effective for the treatment of depression as well as smoking cessation among remotely enrolled adult smokers with depressive symptoms recruited from across the country. Findings support the utility of this intervention approach for addressing the currently unmet public health treatment need for tailored, scalable depression-specific cessation treatments.

## Appendix

Multimedia Appendix 1 Goal2Quit uptake.

Multimedia Appendix 2 Goal2Quit app retention (N=103).

Multimedia Appendix 3 CONSORT-eHEALTH checklist (V 1.6.1).

## Abbreviations

BA: behavioral activation
BDI-II: Beck Depression Inventory-II
CONSORT: Consolidated Standards of Reporting Trials
CPD: cigarettes per day
NRT: nicotine replacement therapy
OR: odds ratio
PPA: point prevalence abstinence
REDCap: Research Electronic Data Capture
TAU: treatment as usual
