# Supplementary material for: Behavioral Activation–Based Digital Smoking Cessation Intervention for Individuals With Depressive Symptoms: Randomized Clinical Trial
Source: J Med Internet Res. 2023 Nov 1;25:e49809. doi: 10.2196/49809 (PMC10652199; doi:10.2196/49809)
Supplement: Multimedia Appendix 1 [file jmir_v25i1e49809_app1.docx]

**Table S1.** Goal2Quit uptake.

| Metric | Mean (SD) | Range |
| --- | --- | --- |
| Total number of sessions | 19.3 (30.1) | 1-189 |
| Average time per session (s) | 177.8 (129.3) | 33-759 |
| Total time spent using Goal2Quit (min) | 33.1 (40.1) | 4.37-201.0 |
| Activities scheduled | 72.8 (112.5) | 1-487 |
| Activities completed | 14.0 (42.5) | 0-315 |
