# Supplementary material for: Behavioral Activation–Based Digital Smoking Cessation Intervention for Individuals With Depressive Symptoms: Randomized Clinical Trial
Source: J Med Internet Res. 2023 Nov 1;25:e49809. doi: 10.2196/49809 (PMC10652199; doi:10.2196/49809)
Supplement: Multimedia Appendix 2 [file jmir_v25i1e49809_app2.docx]

**Table S2.** Goal2Quit app retention (N=103).

| Week following initial app download | Values, n (%) |
| --- | --- |
| Week 1 | 103 (100) |
| Week 2 | 70 (68) |
| Week 3 | 46 (45) |
| Week 4 | 40 (39) |
| Week 5 | 33 (32) |
| Week 6 | 28 (27) |
| Week 7 | 24 (23) |
| Week 8 | 24 (23) |
| Week 9 | 18 (17) |
| Week 10 | 13 (13) |
| Week 11 | 11 (11) |
| Week 12 | 7 (7) |
